# Facile synthesis of ultrathin magnetic iron oxide nanoplates by Schikorr reaction

**DOI:** 10.1186/1556-276X-8-16

**Published:** 2013-01-07

**Authors:** Ming Ma, Yu Zhang, Zhirui Guo, Ning Gu

**Affiliations:** 1 State Key Laboratory of Bioelectronics and Jiangsu Key Laboratory of Biomaterials and Devices, School of Biological Science and Medical Engineering, Southeast University, Nanjing, 210009, People’s Republic of China; 2 The Second Affiliated Hospital of Nanjing Medical University, Nanjing, 210011, People’s Republic of China

**Keywords:** Magnetite nanoplates, Schikorr reaction, Ethylene glycol, Ferrous hydroxide

## Abstract

In this work, a very facile one-pot hydrothermal synthesis approach has been developed for the preparation of ultrathin magnetite nanoplates. The hydrothermal procedure was performed by aging ferrous hydroxide under anaerobic conditions, which is known as Schikorr reaction. Ethylene glycol (EG), which was introduced to the reaction as another solvent, played a critical role in the formation process of these nanoplates. Typically, hexagonal Fe_3_O_4_ nanoplates with a thickness of 10 to 15 nm and a side length of 150 to 200 nm have been synthesized with EG/H_2_O = 1:1 in experiments. Our data suggest that the thickness of Fe_3_O_4_ nanoplates decreases, and the shape of the nanoplate becomes more irregular when the concentration of EG increases. The as-prepared Fe_3_O_4_ nanoplates were highly crystallized single crystals and exhibited large coercivity and specific absorption rate coefficient.

## Background

Magnetite (Fe_3_O_4_) is an attractive material for essential applications such as magnetic storage, ferrofluids, catalysts, chemical sensor, biological assays, and hyperthermia because of its magnetic features combined with nanosize and surface effects [1-9]. To date, a number of nanosized magnetite crystals with a variety of morphologies, such as nanoparticles, nanospheres, hollow spheres, nanorods, nanowires, nanotubes, nanorings, nanopyramids, nano-octahedra, and flowerlike nanostructures, have been prepared by a variety of chemistry-based processing routes, including coprecipitation, thermal decomposition, microemulsion, electrochemical synthesis, and solvothermal or hydrothermal synthesis [10-15]. However, to the best of our knowledge, there are only limited reports concerning the synthesis of ultrathin magnetite nanoplate and its interesting properties. Chen's group synthesized γ-Fe_2_O_3_ nanoplates by a solvothermal process using ethanol as solvent and poly(vinylpyrrolidone) (PVP) as stabilizer, followed by a reduction process to generate Fe_3_O_4_ nanoplates [16]. Xu and coworkers prepared triangular Fe_3_O_4_ nanoplates between two carbon films by pyrolyzing ferrocene and sodium oxalate at 600°C [17].

In this work, we report a facile one-pot hydrothermal approach for the preparation of magnetite nanoplates by the famous Schikorr reaction. Under anaerobic conditions, iron(II) hydroxide can be oxidized by the protons of water to form iron(II,III) oxide and molecular hydrogen. This process is described by the Schikorr reaction [18-20]:

(1) $${3\mathrm{Fe}\left(\mathrm{OH}\right)}_{2}\to {\mathrm{Fe}}_{3}O_{4}\;+\;H_{2}\;+\;{2H}_{2}O$$

The Schikorr reaction usually occurs in the process of anaerobic corrosion of iron and carbon steel in various conditions [21,22]. Herein, this reaction was used to prepare magnetite nanoplates. In addition, ethylene glycol (EG) was introduced to this reaction as another solvent besides H_2_O to adjust the morphology and thickness of the products. In a typical procedure, a FeSO_4_ water solution was added to a H_2_O-EG mixture containing NaOH at a constant rate and under stirring after nitrogen was bubbled through the two solutions for 2 h. When the precipitation was completed, the system was undisturbed and heated to 90°C for 24 h.

## Methods

### Materials

All chemicals used in our experiments were purchased and used as received without further purification. Iron(II) sulfate heptahydrate (FeSO_4_·7H_2_O, 99+%), ethylene glycol (C_2_H_6_O_2_, 99%), and sodium hydroxide (NaOH, 98%) were purchased from Alfa Aesar (Ward Hill, MA, USA). Sulfuric acid (H_2_SO_4_, >92%) was purchased from Shanghai Ling-Feng Chemical Reagent Co., Ltd. (Changshu City, China).

### Synthesis

In the typical synthetic procedure of the Fe_3_O_4_ nanoplates, nitrogen is bubbled through two solutions independently: (a) 54 ml of water-EG mixture containing NaOH to obtain the final concentration of 0.22 M NaOH and (b) 6 ml of FeSO_4_·7H_2_O dissolved in 10^−2^ M H_2_SO_4_ to obtain the final concentration of 2*.*4 × 10^−2^ M. After 2 h, the iron(II) sulfate solution was added to the basic solution at a constant rate and under stirring. When the precipitation was completed, nitrogen was allowed to pass for another 3 min, and the system was undisturbed and heated to 90°C for 24 h in a Teflon autoclave. Aging time was fixed at 24 h in order to reach conditions near equilibrium. At this point, the solution was cooled at room temperature with an ice bath, and the solid was separated by magnetic decantation and washed several times with distilled water.

### Characterization

The morphology and microstructure were characterized using a transmission electron microscope (TEM; JEM-2100, JEOL, Tokyo, Japan) with an accelerating voltage of 200 kV and a Zeiss Ultra Plus field emission scanning electron microscope (SEM; Zeiss, Oberkochen, Germany) with in-lens capabilities, using nitrogen gas and ultrahigh-resolution BSE imaging. X-ray diffraction (XRD) patterns were collected on a Rigaku D/Max 2200PC diffractometer (Rigaku Corp., Tokyo, Japan) with a graphite monochromator and CuKR radiation. X-ray photoelectron spectra (XPS) were recorded on a PHI-5300 ESCA spectrometer (Perkin-Elmer, Waltham, MA, USA). The infrared spectra were recorded on a Thermo Nicolet-5700 Fourier transform infrared spectrometer (FTIR; Thermo Scientific, Logan, UT, USA). The micro-Raman analyses were performed on a Renishaw Invis Reflex (Renishaw, Gloucestershire, UK) system equipment with Peltier-cooled charge-coupled device and a Leica confocal microscope (Leica, Solms, Germany). The magnetic properties were measured at room temperature using a vibration sample magnetometer (7404, LakeShore, Westerville, OH, USA). To investigate the specific absorption rate (SAR) coefficient of the nanoplates, the calorimetric measurements were performed on an alternating current (AC) magnetic field generator (model SPG-10-I, Shenzhen Shuangping, Guangdong, China; 10 kW, 100 to 300 kHz).

## Results and discussion

**Figure 1:**
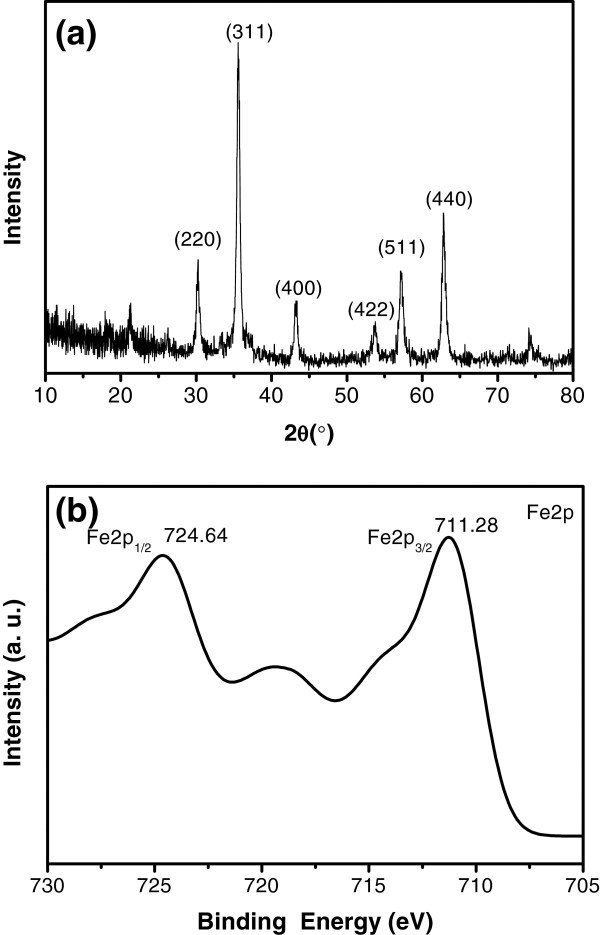
**X-ray diffraction patterns (a) and Fe2*p *XPS patterns of as-synthesized products (EG/H**_**2**_**O = 1:1) (b).**

**Figure 2:**
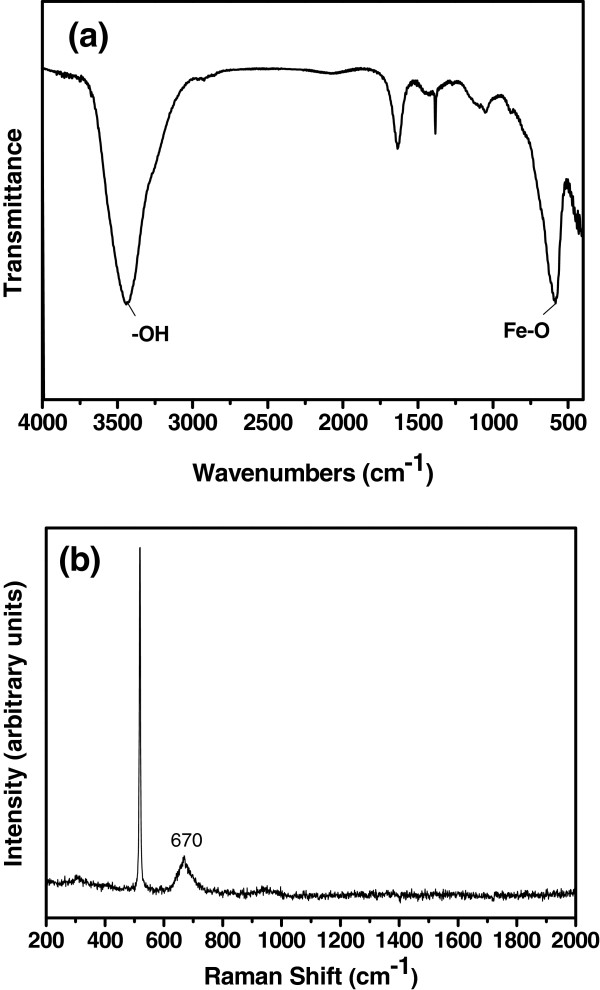
**FTIR (a) and Raman spectra (b) of as-synthesized products (EG/H**_**2**_**O = 1:1).**

The XRD pattern (Figure 1a) of the obtained material proves its crystalline nature of face-centered cubic structure, and the peaks match well with standard Fe_3_O_4_ reflections (JCPDS card no. 86–1354) [23]. XPS was then used to determine the product because XPS is very sensitive to Fe^2+^ and Fe^3+^ cations. The representative XPS spectra (Figure 1b) of the prepared product indicate that the levels of Fe2*p*_3/2_ and Fe2*p*_1/2_ are 711.28 and 724.64 eV. It is in agreement with the literature that the peaks shift to high binding energy and broaden for Fe_3_O_4_ due to the appearance of Fe^2+^(2*p*_3/2_) and Fe^2+^(2*p*_1/2_) [24]. IR and Raman analyses (Figure 2) were employed to further confirm whether the product was magnetite rather than the other oxide or oxyhydroxide of iron. The IR spectra of the product (Figure 2a) display one peak at around 570 cm^−1^; this peak is attributed to the Fe-O functional group of Fe_3_O_4_, whereas α-Fe_2_O_3_ and γ-Fe_2_O_3_ exhibit two or three peaks between 500 and 700 cm^−1^[25,26], which are different from Fe_3_O_4_. Raman spectroscopy is a powerful tool to study the internal structure of molecules and structures. Various iron oxides and oxyhydroxides can be successfully identified using Raman spectroscopy [27]. Figure 2b shows the Raman spectrum of the product dried on Si substrate. Besides the sharp peak at 520 cm^−1^, which is attributed to the Si substrate, the Raman spectrum displays only one peak at 670 cm^−1^, which is the principal Raman band of magnetite [27,28]. The IR and Raman analyses combined with XRD pattern and XPS spectra can confirm the synthesis of Fe_3_O_4_. 

**Figure 3:**
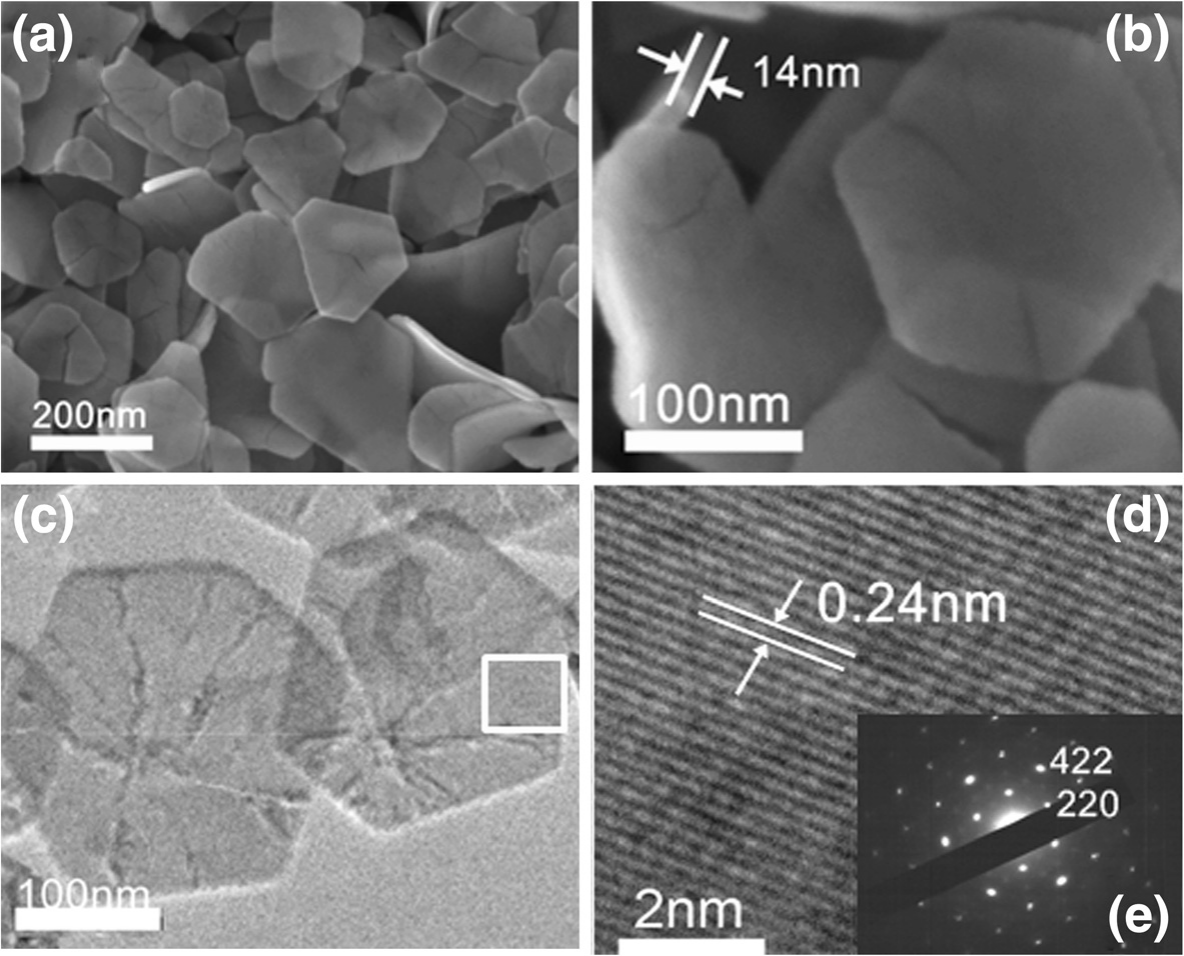
**Low- (a) and high-magnification (b) SEM images of the as-prepared Fe**_**3**_**O**_**4 **_**nanoplates (EG/H**_**2**_**O = 1:1). **The thickness of the nanoplate is about 14 nm. **(c)** TEM image of the same nanoplate sample. **(d)** HRTEM image of the marked area shown in (c). Both the HRTEM image (d) and the SAED pattern **(e)** show that the nanoplate is a single crystal.

Figure 3a shows the SEM image of Fe_3_O_4_ products prepared with EG/H_2_O = 1:1 in the experiment, and it can be seen that the products exhibit a plate-like morphology with a thickness of 10 to 15 nm and a side length of 150 to 200 nm. Most of the nanoplates have hexagonal shapes, and a few are irregular polygons. TEM image of the same sample further reveals that the product consists of plate-shaped structures with a hexagonal outline, as shown in Figure 3c. The corresponding selected area electron diffraction (SAED) pattern (Figure 3e) was obtained directing the incident electron beam perpendicular to one hexagonal facet of an individual nanoplate, and one set of diffraction spots could be indexed as the (220) and (422) reflections, respectively, which demonstrated that the two hexagonal facets were bounded by the {111} facets. It is deduced that the growth of the nanoplates along the [111] direction would be hindered to make the {111} planes as the basal planes of the nanoplates. More detailed information on the nanoplate was acquired using high-resolution TEM (HRTEM). The HRTEM images of the area marked by rectangles are shown in Figure 3d. The lattice fringes observed in the images are about 0.24 nm, which agree well with the separation between the (211) lattice planes of magnetite. The SAED and HRTEM analyses reveal that the as-prepared sample has a cubic structure. 

**Figure 4:**
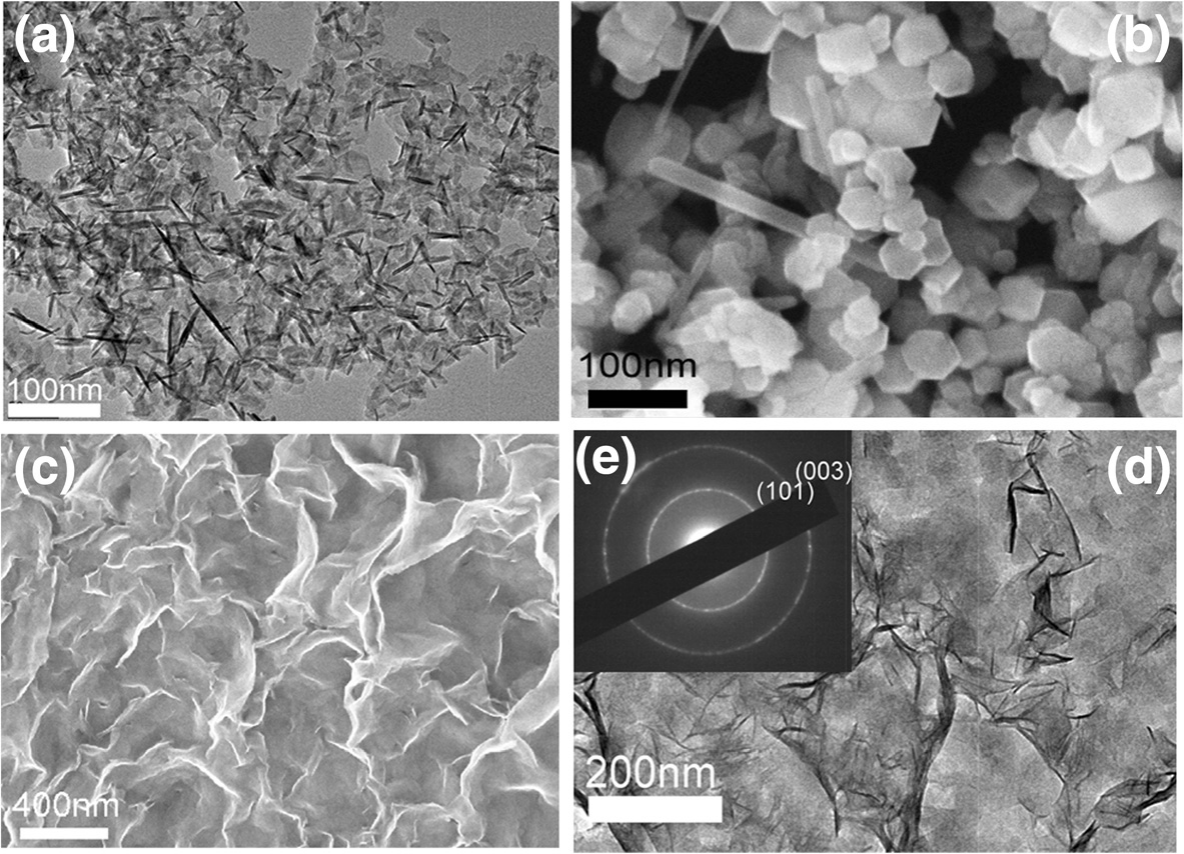
**Fe(OH)**_**2**_**and the as-prepared Fe**_**3**_**O**_**4**_**. (a)** TEM images of Fe(OH)_2_and **(b)** low-magnification SEM images of the as-prepared Fe_3_O_4_obtained in pure aqueous solution. It can be seen that the product is a mixture of polygonal particles and fiber-like particles. **(c)** SEM and **(d)** TEM images and **(e)** the SAED pattern of Fe(OH)_2_ obtained in the EG-H_2_O mixture.

**Figure 5:**
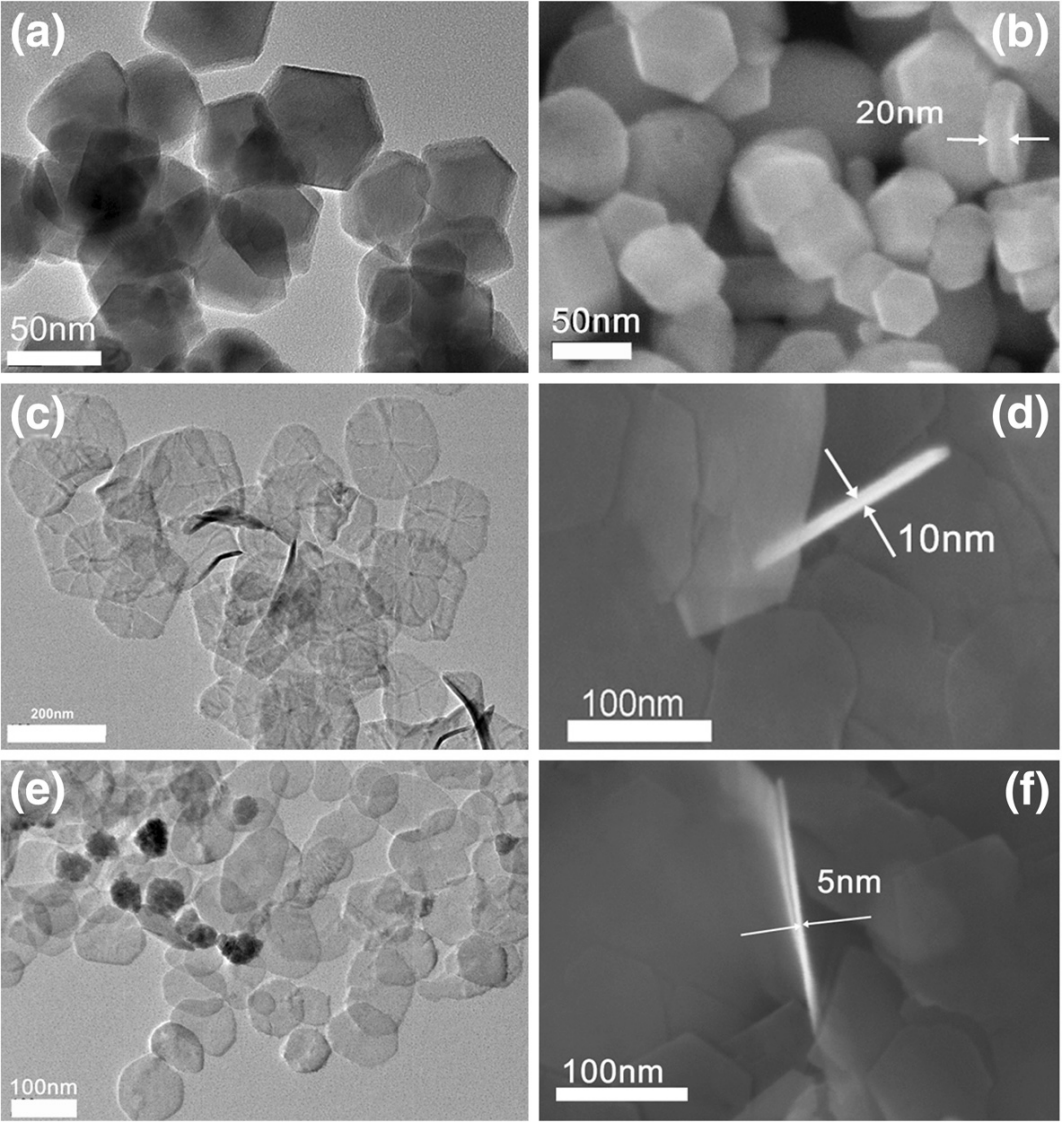
**The Fe**_**3**_**O**_**4 **_**nanoparticles and nanoplates prepared under different conditions. (a)** TEM and **(b)** SEM images of the as-prepared Fe_3_O_4_ nanoparticle (EG/H_2_O = 0:1). **(c)** TEM and **(d)** SEM images of Fe_3_O_4_ nanoplates prepared under the condition of EG/H_2_O = 3:1. The diameter of the nanoplates is about 150 to 200 nm, and the thickness of the nanoplate is about 10 nm. **(e)** TEM and **(f)** SEM images of the Fe_3_O_4_ nanoplates prepared under the condition of EG/H_2_O = 5:1. The diameter is about 80 to 10 nm, and the thickness is about 5 nm.

Ferrous hydroxide (Fe(OH)_2_) is the crucial precursor of the reaction. Ferrous hydroxide has a cadmium iodide structure with a space grouping of P3m1 [29]. Fe atoms occupy only one set of octahedra out of two between the anion layers A and B of the ABAB stacking sequence. The layer structure of ferrous hydroxide makes it tend to form sheet- or plate-shaped crystal. Ethylene glycol is a strong reducing agent with a relatively high boiling point and has been widely used in the polyol process to provide monodispersed fine metal or metal oxide nanoparticles [30-34]. Further studies indicate that the concentration of EG plays an important role in the formation of precursor Fe(OH)_2_ and the end product Fe_3_O_4_ nanoplate. In the chemical reaction scheme, ferrous hydroxide is typically prepared by adding FeSO_4_ water solution to a NaOH solution. We investigated the morphology and structure of the as-obtained precipitate by TEM, SEM, and SAED, respectively. When the solvent of the whole system is only water (none of EG), a dark-green precipitate is produced immediately after the FeSO_4_ solution is dropped into excessive NaOH solution. In contrast to pure aqueous solution, the precipitate of ferrous hydroxide in the H_2_O-EG mixture solution was white at the beginning and turns green then dark-green gradually. The precipitate of ferrous hydroxide obtained in pure aqueous solution is also known as ‘green rust’ in the crystal lattice of which iron(II) ions are easily substituted by iron(III) ions produced by its progressive oxidation [35-37]. However, the oxidation process is inhibited in the H_2_O-EG mixture solution because of the reducing power of EG. All forms of green rust are more complex and variable than the ideal iron(II) hydroxide compound. TEM images of the precipitate (Figure 4a) obtained in pure aqueous solution show that there are two kinds of products at least; one of them is a very thin nanoplate with a diameter of about 50 nm, and the other is a needle-shaped nanoparticle. TEM and SEM images (Figures 4b and 5a,b) of the end product of this precipitate after aging for 24h in 90°C show that the obtained product is a mixture of polygonal particles and fiber-like particles. The sizes of the polygonal particles are about 50 to 100 nm. However, no rod-like or fiber-like nanoparticles can be found in the TEM and SEM images of the as-obtained ferrous hydroxide precipitate (Figure 4c,d) in the H_2_O-EG mixture solution. Ferrous hydroxide obtained in the H_2_O-EG mixture solution forms a large-scaled film rather than plate-like and rod-like nanoparticles in pure aqueous solution. Also, according to its SAED pattern (Figure 4e), the ferrous hydroxide film has a polycrystalline structure. TEM and SEM images of the Fe_3_O_4_ nanoplate obtained in the EG-H_2_O mixture solution with the ratio of EG/H_2_O = 3:1 and 5:1 are shown in Figure 5c,d,e,f. It can be seen that the thickness of the Fe_3_O_4_ nanoplates decreases, and the shape of the nanoplate becomes more irregular when the concentration of EG increases. From the analysis of the above experiments, it is obvious that the addition of EG affects the formation of Fe_3_O_4_ nanoplate. 

**Figure 6:**
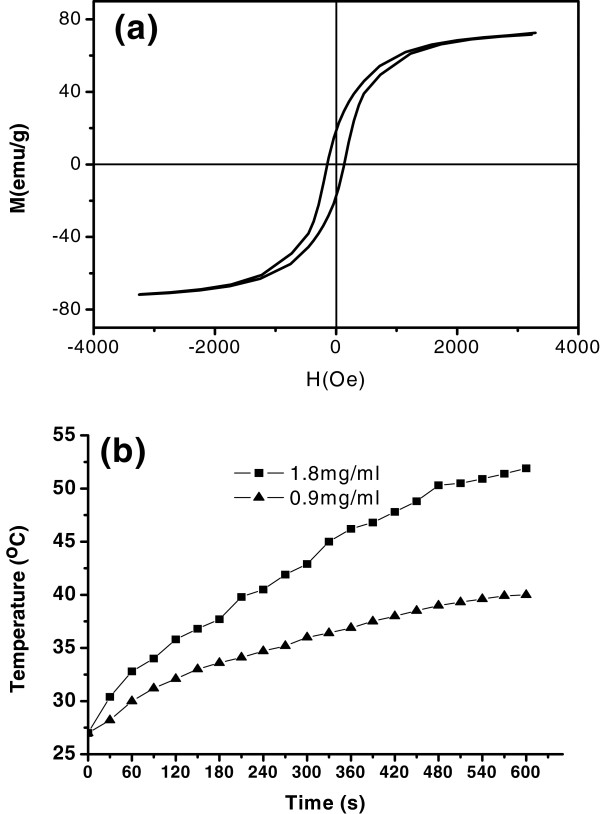
**The Fe**_**3**_**O**_**4 **_**nanoplates obtained in EG/H**_**2**_**O = 1. (a)** Magnetic hysteresis loop measured at room temperature for the Fe_3_O_4_nanoplates (EG/H_2_O = 1:1). **(b)** Temperature versus time curves of Fe_3_O_4_ nanoplates (EG/H_2_O = 1:1) dispersed in aqueous solution under an AC magnetic field (0.95 kA/m, 176 kHz).

The typical magnetic hysteresis loop of the Fe_3_O_4_ nanoplates obtained in EG/H_2_O = 1 is depicted in Figure 6a. It exhibits a ferromagnetic behavior with saturation magnetization (*M*_s_), remanent magnetization (*M*_r_), and coercivity (*H*_c_) values of *ca*. 71.6 emu/g, 18.4 emu/g, and 152.2 Oe, respectively. It is well known that the saturation magnetization and the coercive field of bulk Fe_3_O_4_ are about 85 to 100 emu/g and 115 to 150 Oe, respectively [38]. From the results, it can be seen that the saturation magnetization value is lower than that of bulk Fe_3_O_4_. The reduced value might be due to the spin canting of surface Fe atoms [39-41]. Compared with bulk magnetite, the as-prepared nanoplates exhibit enhanced coercivity. The enhanced coercivity may be attributed to the large sharp anisotropic nature of the nanoplates which represents the barrier for particle remagnetization [42]. According to our earlier study, hysteresis loss of magnetite in AC magnetic field with low frequency and high amplitude can be assumed to be proportional to coercivity [43]. Thus, the as-prepared Fe_3_O_4_ nanoplates with enhanced coercivity may have enhanced hysteresis loss in AC magnetic field. We investigated the SAR coefficient of the Fe_3_O_4_ nanoplates by time-dependent calorimetric measurements. The frequency and amplitude of the magnetic field are 180 kHz and 0.95 kA/m, respectively. The temperature versus time curves of Fe_3_O_4_ nanoplate-based ferrofluids are shown in Figure 6b. According to the curves, the SAR for the nanoplates was calculated using the following equation [43,44]:

$$\mathrm{SAR}=C\frac{\mathrm{\Delta T}}{\mathrm{\Delta t}}\frac{1}{m_{\mathrm{Fe}}}$$

where *C* is the sample-specific heat capacity which is calculated as a mass weighted mean value of magnetite and water. For magnetite, *C*_mag_ = 0.937 J/g K, and for water *C*_wat_ = 4.18 J/g K. Δ*T*/Δ*t* is the initial slope of the time-dependent temperature curve. *m*_Fe_ is the iron content per gram of the Fe_3_O_4_ suspension solution. The obtained SAR value is 253.7 ± 27.3 W/g. This value is very high compared to the reported values of Fe_3_O_4_[43,45] and indicates that this material is likely to be very suitable for application in tumor magnetic hyperthermia.

## Conclusions

In summary, ultrathin single-crystalline Fe_3_O_4_ nanoplates can be synthesized facilely on a large scale by a hydrothermal route of Schikorr reaction. The experimental results showed that the concentration of EG played a key role in the information and adjustment of the thickness of the nanoplates. The as-prepared Fe_3_O_4_ nanoplates are highly crystallized single crystals. Also, Fe_3_O_4_ nanoplates are ferromagnetic at room temperature and exhibit large coercivity and specific absorption rate coefficient under external alternating magnetic field.

## Competing interests

The authors declare that they have no competing interests.

## Authors’ contributions

MM conceived, designed, and carried out the experiments, analyzed the data, and wrote the paper. YZ and ZG provided comments/suggestions. NG guided the research. All authors discussed the results, and read and approved the final manuscript.
